# Pomegranate Peel Extract Mitigates Diarrhea-Predominant Irritable Bowel Syndromes via MAPK and NF-κB Pathway Modulation in Rats

**DOI:** 10.3390/nu16223854

**Published:** 2024-11-11

**Authors:** Yannan Zhang, Sijuan Huang, Shuai Zhang, Zhihui Hao, Jianzhong Shen

**Affiliations:** 1 National Key Laboratory of Veterinary Public Health Security, College of Veterinary Medicine, China Agricultural University, Beijing 100193, China; b20193050423@cau.edu.cn (Y.Z.); b20223050398@cau.edu.cn (S.H.); b20243050538@cau.edu.cn (S.Z.); 2 Key Biology Laboratory of Chinese Veterinary Medicine, Ministry of Agriculture and Rural Affairs, Beijing 100193, China

**Keywords:** diarrheal irritable bowel syndrome (IBS-D), pomegranate peel extract (PPE), inflammation responses, oxidant stress, intestinal barrier

## Abstract

Background: Diarrhea-predominant irritable bowel syndrome (IBS-D) is a common chronic functional gastrointestinal disorder that causes diarrheal and intestinal barrier disruptions. Although pomegranate peel extract (PPE) has been reported for the treatment of diarrheal and intestinal inflammation, its effectiveness and mechanisms specifically for the treatment of IBS-D remain unknown. Objectives: This study aimed to explore the therapeutic effect of PPE on IBS-D and elucidate its underlying mechanisms. Methods: A high-fat diet, restraint stress, and senna gavage were combined to establish a rat model mimicking IBS-D, to evaluate the therapeutic effects of PPE. Network pharmacology analysis, serum medicinal chemistry, and transcriptomics were employed to investigate potential downstream signaling pathways. Findings were further validated through molecular docking and Western blot analysis. Results: The findings revealed that PPE significantly improved the symptoms of IBS-D, ameliorated intestinal inflammation, and promoted intestinal barrier function. The target genes in the MAPK and NF-κB signaling pathways were significantly enriched and down-regulated. Molecular docking and Western blot assays were performed to verify that PPE had a high affinity for the protein candidates in these pathways, and significantly down-regulated the expression of p-IκB, p-p65, p-JNK, p-p38, and p-ERK1/2. Conclusions: The present study is the first to demonstrate that PPE alleviates diarrheal and intestinal damage in IBS-D, potentially by inhibiting MAPK and NF-κB signaling pathways. These findings suggest that PPE may provide a novel therapeutic option for IBS-D.

## 1. Introduction

IBS-D is a common, chronic functional gastrointestinal disorder characterized by diarrhea, abdominal pain, and visceral hypersensitivity, typically occurring without notable alterations in organ structure [1]. Due to its high prevalence, IBS-D severely impairs patients’ quality of life and imposes a significant burden on families and communities [2,3]. IBS-D is a complex condition with multiple contributing factors, including gastrointestinal neurotransmitter secretion disorders, intestinal barrier dysfunction, disruptions in the intestinal microbiota, stress, genetic factors, and other potential contributors [4,5,6,7]. Clinical treatments encompass lifestyle modifications, dietary changes, medication adjustments, fecal microbiota transplantation, probiotic supplementation, and antidepressant therapy [8]. Additionally, exposure to cold stress has been associated with an increased incidence of IBS-D [9,10]. In preclinical study, the IBS-D animal model is commonly induced with senna, a laxative that stimulates intestinal peristalsis, mimicking the diarrhea symptoms observed in human IBS-D patients [11]. This is often combined with cold stress and restraint stress, as these methods are widely recognized as effective for simulating key aspects of IBS-D. However, despite these models, the pathophysiological mechanisms of IBS-D are not fully understood, and current therapeutic approaches lack clear targets.

Inflammation and oxidative stress play pivotal roles in the pathogenesis of intestinal diseases [12]. Recent studies have highlighted that intestinal barrier dysfunction contributes to the development of IBS-D [13]. Specifically, intestinal epithelial cells form a protective barrier and are crucial for initiating an appropriate mucosal immune response following infection or injury [14]. Ramakrishnan et al. demonstrated that non-classical NF-κB signaling intestinal epithelial cells are an important homeostatic pathway regulating intestinal inflammatory responses [15]. Additionally, oxidative stress can trigger an inflammatory response [16]. An imbalance between reactive oxygen species (ROS) production and antioxidant defenses leads to oxidative stress, which activates inflammation-associated transcription factors, culminating in intestinal inflammation [17,18,19]. Mitogen-activated protein kinases (MAPK), including c-Jun N-terminal kinase (JNK), p38 mitogen-activated protein kinase (p38 MAPK), and extracellular signal-regulated kinase (ERK), are sequentially activated and regulate a wide range of important cellular physiological and pathological processes, such as intestinal inflammation [20]. It has been demonstrated that certain medications can modulate the MAPK-ERK signaling pathway to alleviate the visceral hypersensitivity of IBS-D, thereby improving the clinical symptoms such as abdominal pain and diarrhea [21]. Therefore, enhancing the intestinal barrier by modulating the NF-κB and MAPK signaling pathways may represent a promising strategy for managing IBS-D.

PPE, derived from the peel of the pomegranate, exhibits potent anti-inflammatory, antibacterial, antioxidant, insecticidal, anticancer, and anti-diarrheal properties, which are attributed to its high concentrations of polyphenols, tannins, and anthocyanins [22]. Although PPE was traditionally considered agricultural waste with low utilization value, recent study has demonstrated that pomegranate peels can serve as effective natural food preservatives by controlling nutrient release [23]. When applied during the storage of fresh fruit, PPE provides a healthier and more sustainable processing method [24]. Furthermore, PPE holds significant potential as raw material for producing biofuels and various biochemical products, emphasizing its value beyond conventional applications [25]. In addition to these applications, a recent study has shown that the pomegranate peel–marmalade medicinal pair alleviates DSS-induced colitis via the IL-6/STAT3/SOCSP3 pathway [26]. Furthermore, it also mitigates colonic damage and bacterial translocation in Citrobacter-induced colitis in mice [27]. Yin et al. demonstrated that PPE enhances bacterial clearance in mice by promoting phagocytosis by macrophages through activation of NF-κB and MAPK pathways, as well as up-regulation of C-type lectin receptor expression [28]. Akuru reported that PPE effectively scavenges hydrogen peroxide and free radicals such as DPPH, superoxide ions, and hydroxyl and peroxyl radicals, thereby preventing oxidative damage to cells [29]. A recent study investigated for the first time how pomegranate peel components ellagic tannins (ETs), ellagic acid (EA), and urolithins (Uros) may improve disease by modulating the brain–gut axis and showed that these biomolecules may contribute to the functional stabilization of the gut barrier or the blood–brain barrier, as well as the prevention or treatment of inflammation-related diseases [30]. The study conducted by Duarte et al. reveals the role of PPE in maintaining intestinal health, suggesting that this extract may ameliorate high-fat diet-induced changes in intestinal metabolism by modulating intestinal flora [31]. These findings highlight the potential of PPE in combating intestinal disorders. However, the efficacy of PPE in the treatment of IBS-D and the underlying mechanisms remains unclear.

Therefore, in the present study, we investigated the chemical composition of PPE using ultra-performance liquid chromatography-tandem mass spectrometry (UPLC-MS/MS) techniques and established a rat model of IBS-D by a high-fat diet, restraint stress, and senna gavage. Furthermore, we assessed the symptomatic and diarrhea-relieving effects of PPE on IBS-D, in addition to its anti-inflammatory effects, antioxidant capacity, and intestinal protective effects, by monitoring the corresponding indices associated with IBS-D. In addition, we analyzed the underlying mechanisms using network pharmacology, transcriptomics, and ultimately molecular docking, followed by experimental validation, to comprehensively elucidate the underlying mechanisms.

## 2. Materials and Methods

### 2.1. Preparations of PPE and Senna Leaf Extract

Dried pomegranate peel was purchased from Sanyuan Longsheng Biotechnology Limited Liability Company (Xianyang, China) and crushed into a coarse powder, passing through a 30-mesh sieve. The powder was then extracted with 60% ethanol at 60 °C three separate times, followed by filtration through a 60-mesh sieve. The filtrate was concentrated under reduced pressure until it reached a specific gravity of 1.2. Subsequently, the concentrate was spray-dried, crushed, and sieved through an 80-mesh sieve to obtain the final PPE.

Senna leaf (Tongrentang Pharmacy Co., Ltd., Beijing, China) was soaked in hot water for 12 h, and the process was repeated once. The filtrates were then combined and concentrated to a final concentration of 1.0 g/mL, which was used for subsequent animal testing.

### 2.2. Animals

The animals used in the study were 5~6-week-old male SD rats, weighing between 180~220 g. In the present study, all rats were purchased from SPF (BEIJING) Biotechnology Co., Ltd. (Beijing, China) (SCXK(Jing)2019-0010). All rats were housed in a room maintained at a constant temperature of 23~25 °C, with a 12 h light/dark cycle and had free access to food and water throughout the study. All the experimental procedures in the study were approved by the Animal Welfare and Ethics Review Committee of Experimental Animals of China Agricultural University (Approval number: AW01203202-2-1; approval date: 10 February 2023).

### 2.3. Establishment of the IBS-D Model

The IBS-D model was established with minor modifications based on previous methods [32,33], and the Mead’s resource equation was employed to guide our sample size selection. Specifically, after a week of adaptation, rats were administered 2 mL of lard (Guanshengyuan, Shanghai, China) and exposed to cold water stress (4 cm depth) for 8 h daily over the course of 1 week. Subsequently, the rats received 3 g/kg senna extract via gavage and were subjected to 1 h of daily restraint stress, which began 1 h after senna administration, for 2 weeks to induce IBS-D.

### 2.4. Grouping and Dosing

After successful evaluation of the IBS-D model using the AWR score and the Bristol fecal score, 24 IBS-D rats were randomly divided into four groups (n = 6 per group): the model group (administered sterile water), the pinaverium bromide (PVB) group (13.5 mg/kg), the high-dose group (PPE-H, 400 mg/kg) and the low-dose group (PPE-L, 200 mg/kg). All treatment were administered daily for 1 week. Additionally, a group of healthy rats (n = 6) from the same batch served as the control group, receiving a regular diet and an equivalent volume of sterile water without any modeling-related interventions. At the end of the experimental period, all rats were anesthetized using isoflurane and humanely euthanized. Fresh fecal, serum, and tissue samples were then collected for subsequent analysis.

### 2.5. Abdominal Withdrawal Reflex (AWR), Bristol Fecal Score and Fecal Water Content

The severity of visceral pain in response to colorectal distension was assessed using the AWR scores. The procedure involved measuring the volume of water injected into the colon, and this measurement was repeated three times. Successful model establishment was confirmed when the AWR score reached 2 and the Bristol fecal score reached 5 [34]. Detailed criteria for AWR scoring can be found in the Supplementary Materials, and the Bristol stool scoring system is presented in Table S2. Additionally, the fecal water content was assessed both after model establishment and following PPE administration [35].

### 2.6. Effects on Colonic Tissue Histology

The colon tissue was fixed in 4% paraformaldehyde (Citotest, Haimen, China), gradually dehydrated using a series of ethanol concentrations, and embedded in paraffin. Sections were cut from the paraffin block using a microtome, followed by deparaffinization, hydration, and staining with hematoxylin and eosin (H&E) or alcian blue-periodic acid Schiff stain (AB-PAS) to observe the pathological changes and goblet cell changes in colon tissue. The stained sections were then observed under an optical microscope (DM4B, Leica, Wetzlar, Germany).

### 2.7. Transmission Electron Microscopic (TEM) Observation

A 1 mm^3^ piece of colon tissue was immediately placed in precooled 2.5% glutaraldehyde for at least 4 h at 4 °C, rinsed three times (10 min each) with phosphate buffered saline (PBS), and then fixed with 1.0% osmic acid at room temperature. Ultrathin sections were prepared after ethanol dehydration in a graded series, followed by acetone dehydration (10 min at each concentration), and embedding in epoxy resin. The sections were then stained with uranyl lead and observed for the ultrastructure of colonic epithelial cells using a Hitachi H-7650 transmission electron microscope (Tokyo, Japan).

### 2.8. Enzyme-Linked Immunosorbent Assay (ELISA)

The serum and colonic levels of MPO, IL-1β, IL-6, IL-10, and TNF-α were assessed with ELISA kits (Shanghai Enzyme Linked Biotechnology Co., Ltd., Shanghai, China). The colonic antioxidant capacity was determined by T-AOC, MDA, CAT, and GSH-Px commercial assay kits (Nanjing Jiancheng Biological Technology Institute, Nanjing, China). The tests were performed following the manufacturer’s protocol.

### 2.9. Real-Time Polymerase Chain Reaction

Total RNA was isolated from colon tissue with the total RNA extraction kit (R1200, Solarbio, Beijing, China), and the RNA was quantified using a Nanodrop 2000 spectrophotometer (Thermo Fisher Scientific, Hampton, NY, USA). Then, it was converted into cDNA using a reverse transcription kit (K1961, Thermo Scientific, Waltham, MA, USA). The qRT-PCR experiment was performed using SYBR qPCR Master Mix (Q312, Vazyme, Nanjing, China). The 2^−ΔΔCT^ method, with GAPDH as the internal reference gene, was used to evaluate the relative expression of the target genes. The gene-specific primer sequences were synthesized by Sangon Biotech Co., Ltd. (Shanghai, China) and shown in Table 1.

### 2.10. Western Blotting (WB)

Total proteins were extracted from colon tissue using radioimmunoprecipitation assay (RIPA) (Solarbio, Beijing, China) buffer containing PMSF (1:100, Solarbio, Beijing, China) supplemented with protease and phosphatase inhibitors (1:100, Aladdin, Shanghai, China) on ice, followed by quantification using the BCA method (Thermo Scientific, Waltham, MA, USA). After quantification, protein samples (30 μg) were separated by sodium dodecyl sulfate-polyacrylamide gel electrophoresis (SDS-PAGE) and transferred to polyvinylidene difluoride (PVDF) membranes (Millipore, Merck, Darmstadt, Germany). The membranes were then blocked with 5% skim milk before being hybridized with specific antibodies. The primary antibodies in this study were anti-Occludin (27260-1-AP, Proteintech, Wuhan, China), anti-Claudin1 (13050-1-AP, Proteintech, Wuhan, China), anti-Claudin2 (26912-1-AP, Proteintech, Wuhan, China), anti-ZO-1(21773-1-AP, Proteintech, Wuhan, China) (1:1000 dilution), anti-erk1/2 (#4695, Cell Signaling Technology, Danvers, MA, USA), anti-p-erk1/2 (#4370, Cell Signaling Technology, Danvers, MA, USA), anti-JNK (#9252, Cell Signaling Technology, Danvers, MA, USA), anti-p-JNK (#4668, Cell Signaling Technology, Danvers, MA, USA), anti-P38 (#8690, Cell Signaling Technology, Danvers, MA, USA), anti-p-P38 (#4511, Cell Signaling Technology, Danvers, MA, USA), anti-P65 (#8242, Cell Signaling Technology, Danvers, MA, USA), anti-p-P65 (#3033, Cell Signaling Technology, Danvers, MA, USA), anti-IκBα (#4814, Cell Signaling Technology, Danvers, MA, USA), anti-p-IκBα (#2859, Cell Signaling Technology, Danvers, MA, USA) (1:1000 dilution), and anti-β-actin (bs-0061R, Bioss, Beijing, China) (1:5000 dilution) at 4 °C overnight. After five washes by TBST buffer solution (T1082, Solarbio, Beijing, China), protein bands were visualized using a chemiluminescence detection kit (PK10003, Proteintech, Wuhan, China), and the resulting images were analyzed with ImageJ software (v.2.3.0/1.53f).

### 2.11. Serum Pharmacochemistry Analysis

Male SD rats were gavaged with PPE at a dose of 400 mg/kg, twice daily for 5 days. Blood samples were collected before and 15 min, 30 min, 45 min, 1 h, 2 h, 4 h, 6 h, 8 h, 12 h, 24 h, 36 h, and 48 h after the last administration. After resting for 30 min, the blood samples were centrifuged at 4000 rpm and 4 °C for 5 min. The serum from different time points was stored at −80 °C until later analysis. Plasma sample processing and mass spectrometry conditions are provided in Supplementary Materials.

### 2.12. Network Pharmacology Prediction

The identified PPE components were analyzed by UPLC-Q-TOF-MS (detailed methodology in the Supplementary Materials), and target prediction was obtained from the TCMSP database. IBS-D-related targets were collected from OMIM, Drugbank, and TTD databases. By cross-referencing targets associated with PPE and IBS-D, potential active targets of PPE for the treatment of IBS-D were identified. Next, overlapping targets were selected for protein–protein interaction (PPI) analysis by the STRING platform for further visualization. Core targets were obtained by screening degree centrality (DC) and betweenness centrality (BC) by Cytoscape software (v.3.7.2). Furthermore, Kyoto Encyclopedia of Genes and Genomes (KEGG) analyses were performed, and the results were visualized through the DAVID database. From these analyses, the top 20 KEGG-ranked terms were selected for further study.

### 2.13. Molecular Docking

The three-dimensional structure files of proteins and components were obtained from the RCSB Protein Data Bank (https://www.rcsb.org/, accessed on 15 February 2024) and PubChem (https://pubchem.ncbi.nlm.nih.gov, accessed on 15 February 2024), respectively. Molecular docking was conducted using AutoDock Vina software (v.1.5.7), after replacing water molecules with polar hydrogen atoms in the structures. The docking results were assessed based on binding free energy, with values less than −5 kcal/mol indicating significant binding affinity. The optimal docking configurations were visualized using PyMOL 2.5 software.

### 2.14. Transcriptomic Analysis

Colon tissues were promptly harvested and immediately stored on ice. Total RNA extraction was performed using TRIzol Reagent, and RNA quantification was carried out using the ND-2000 spectrophotometer (Nanodrop Technologies, Wilmington, NC, USA). Only RNA samples that met that the quality control criteria (OD260/280 ratio between 1.8 and 2.2, and OD260/230 ratio ≥ 2.0) were selected for further processing. High-quality RNA was utilized to construct sequencing libraries, which were subsequently sequenced using the Illumina NovaSeq 6000 platform (Shanghai Majorbio Biopharm Biotechnology Co., Ltd., Shanghai, China). Differential gene expression (DEG) analysis was performed using the DESeq2 software package (v.1.23.10). Gene Ontology (GO) and Kyoto Encyclopedia of Genes and Genomes (KEGG) analysis were conducted using Goatools and KOBAS. Genes with a |log2(fold change)| ≥ 2 and a *p*-value ≤ 0.05 were considered differentially expressed. Statistical significance was determined with a corrected *p*-value threshold of less than 0.01.

### 2.15. Statistical Analysis

Statistical analysis was conducted using GraphPad Prism 7.0 software. Data were showed as mean ± SD. Comparison between groups was assessed by one-way analysis of variance (ANOVA) followed by Tukey’s multiple comparison test. Statistical significance was defined as *p* < 0.05.

## 3. Results

### 3.1. Identification of Major Components of PPE

**Figure 1:**
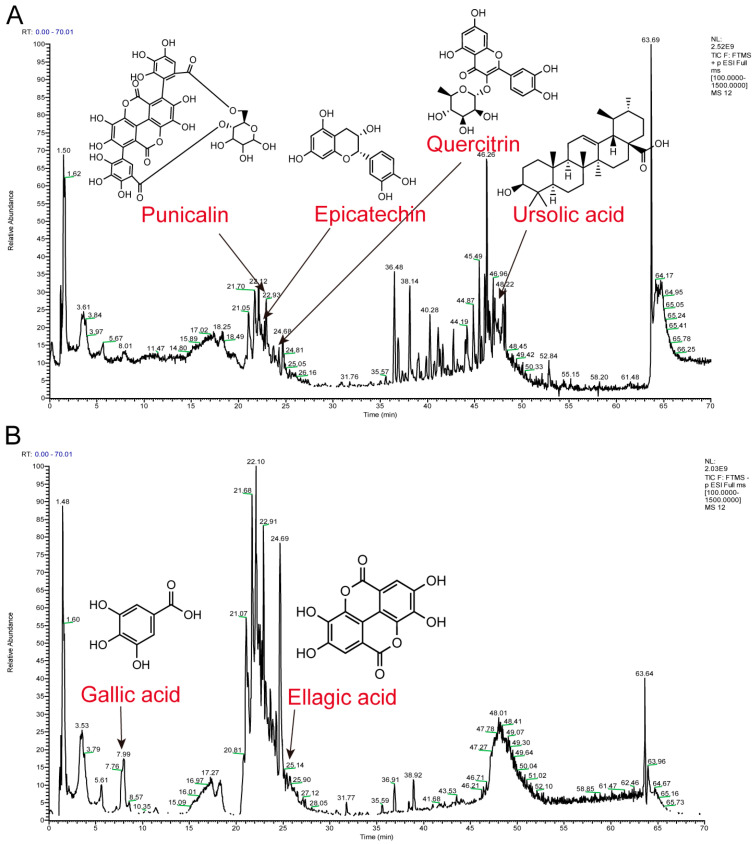
Identification of chemical components of PPE. (**A**) Positive ion mode, (**B**) Negative ion mode.

We characterized the main components of PPE extract by UPLC-Q-Exactive Orbitrap MS/MS (Figure 1 and Table S1), which identified a total of 189 components (Table S3). These included 35 flavonoids, 17 organic acids, 12 amino acids, 11 fatty acids, 11 terpenoids, 10 phenols, 8 lignans, 7 glycosides, 7 amides, 6 esters, 4 nucleosides, 4 vitamins, 3 alkaloids, 3 hormones, 3 amino alcohols, 3 aromatic aldehydes, and 43 others. According to the report, the compounds that may play a key therapeutic role among these included ellagic acid, gallic acid, quercitrin, punicalin, ursolic acid, and epicatechin, with detailed information provided in Table 2. These results highlight the complexity and diversity of compounds present in PPE, which may contribute to its therapeutic properties.

### 3.2. PPE Significantly Relieves Symptoms of IBS-D and Suppresses Inflammation and Oxidative Stress in the Intestine

**Figure 2:**
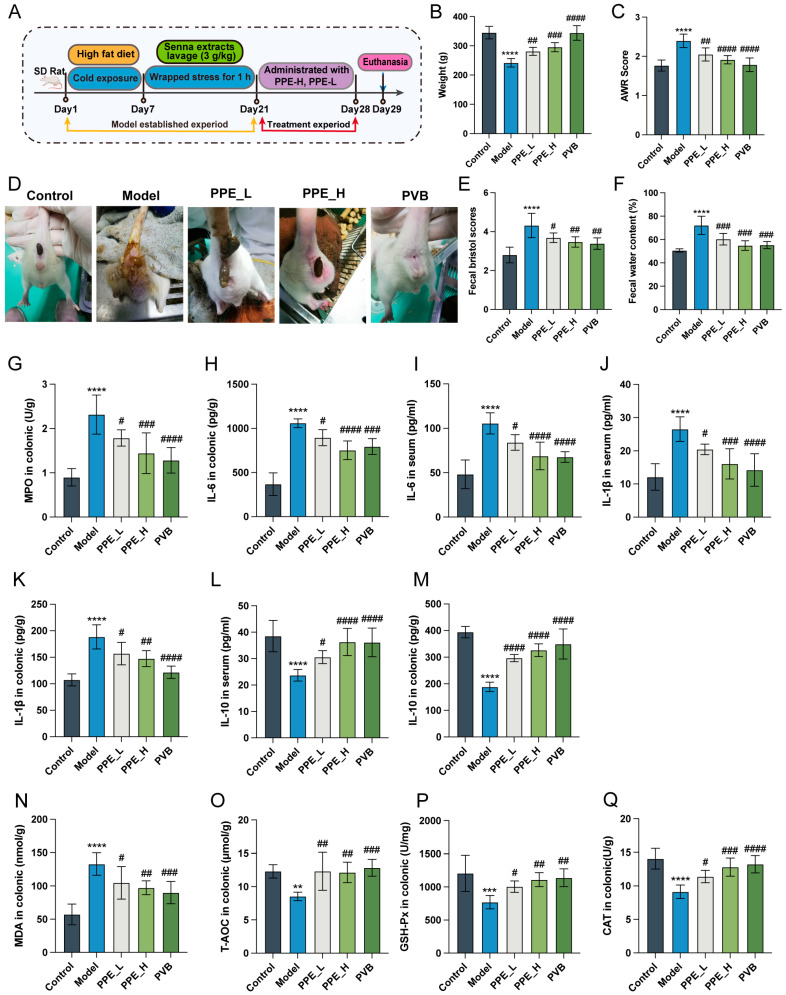
Protective effect of PPE on IBS-D rats (n = 6). (**A**) Overview of the experimental procedure involving PPE treatment for IBS-D. (**B**) Changes in body weight across groups following administration of PPE. (**C**) AWR scores for each experimental group. (**D**) Diarrheal symptoms observed in rats across groups at the conclusion of PPE treatment. (**E**) Bristol stool scores for fecal samples from each group. (**F**) Fecal water content across the groups. (**G**) Levels of MPO in colonic tissues. (**H**–**M**) Levels of IL-6, IL-1β and IL-10 in colon tissue and serum. (**N**–**Q**) Detection of MDA, T-AOC, GSH-Px, and CAT levels in colon. ** *p* < 0.01, *** *p* < 0.001, **** *p* < 0.0001 compared to the control group, # *p* < 0.05, ## *p* < 0.01, ### *p* < 0.001, #### *p* < 0.0001 compared to the model group.

The IBS-D model was established through a combination of a high-fat diet, senna gavage, and restraint stress (Figure 2A) and subsequently treated with PPE. Symptoms associated with IBS-D were evaluated, revealing that PPE administration significantly alleviated diarrhea, as shown by significant reductions in weight loss (Figure 2B), AWR score (Figure 2C), diarrheal situation, fecal Bristol score, and fecal water content in IBS-D rats (Figure 2D–F). Furthermore, PPE treatment led to decreased levels of inflammatory cytokines (MPO, IL-6, and IL-1β) in both serum and colon tissues (Figure 2G–K). In contrast, the anti-inflammatory factor IL-10 was significantly elevated following PPE treatment (Figure 2L,M). These findings indicate that PPE administration effectively reduced intestinal inflammation in IBS-D rats. Moreover, PPE treatment enhanced the antioxidant capacity in IBS-D rats by modulating oxidative stress indices with oxidative stress, such as MDA, T-AOC, GSH-Px, and CAT (Figure 2N–Q), thereby indicating enhanced self-protective mechanisms. Overall, these results demonstrate that PPE treatment alleviated the symptoms of IBS-D through regulation of inflammatory cytokine levels and enhancement of antioxidant capacity.

### 3.3. PPE Facilitated the Integrity of Intestinal Barrier

**Figure 3:**
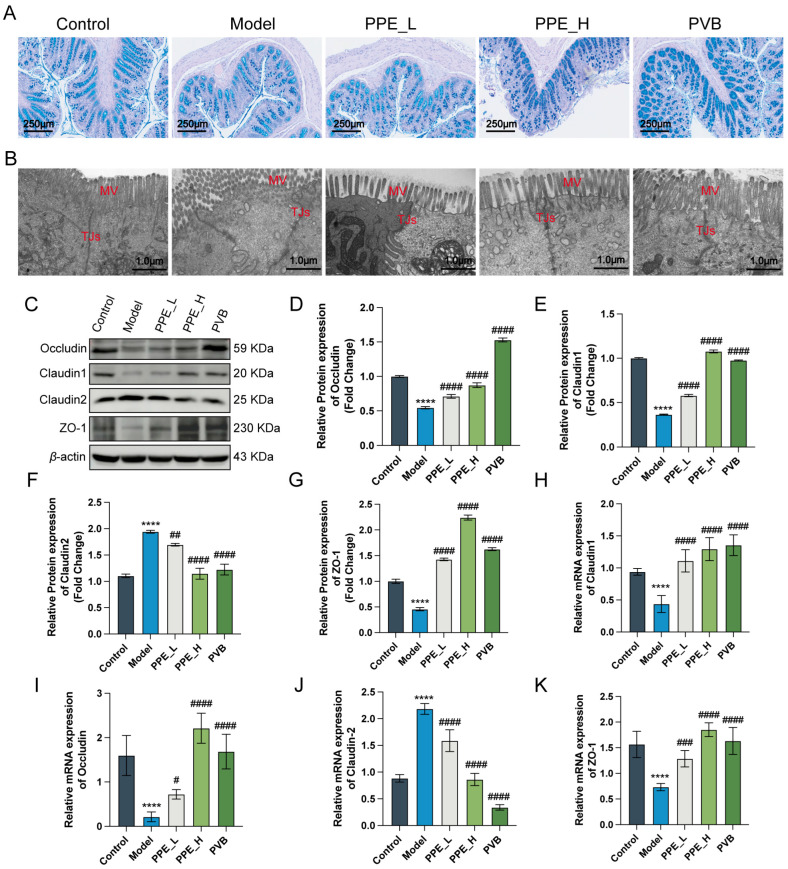
Enhancing effect of PPE on the intestinal barrier of IBS-D rats. (**A**) AB-PAS staining illustrating the protective effect of PPE on goblet cells in IBS-D rats. (**B**) TEM showing the protective effect of PPE on microvilli and tight junctions in IBS-D rats. (**C**) Western blot analysis of protein expression levels for Occludin, claudin-1, claudin-2, and ZO-1. (**D**–**G**) Quantitative analysis of protein expression levels in each group. n = 3. (**H**–**K**) Detection of mRNA levels for the aforementioned proteins in each group, n = 6. **** *p* < 0.0001 compared to the control group, # *p* < 0.05, ## *p* < 0.01, ### *p* < 0.001, #### *p* < 0.0001 compared to the model group.

To assess the impact of PPE on the intestinal barrier in IBS-D rats, we employed AB-PAS staining and transmission electron microscopy (TEM) to investigate the structural integrity of colon tissues. Our results revealed that PPE administration significantly increased the population of goblet cells and conferred protection to microvilli from damage compared to the model group (Figure 3A,B). Furthermore, we evaluated the expression levels of tight junction proteins, especially Occludin, Claudin1, Claudin2, and ZO-1. Our analysis indicated that PPE treatment notably elevated the expression of Occludin, Claudin1, and ZO-1, while concurrently decreasing the expression of Claudin2 (Figure 3C–G). These observations were further corroborated at the mRNA level (Figure 3H–K). Collectively, these findings suggested that PPE plays a significant role in preserving the integrity of the intestinal barrier.

### 3.4. Identification of Bioactive Components and Signaling Pathway Prediction of Network Pharmacology

**Figure 4:**
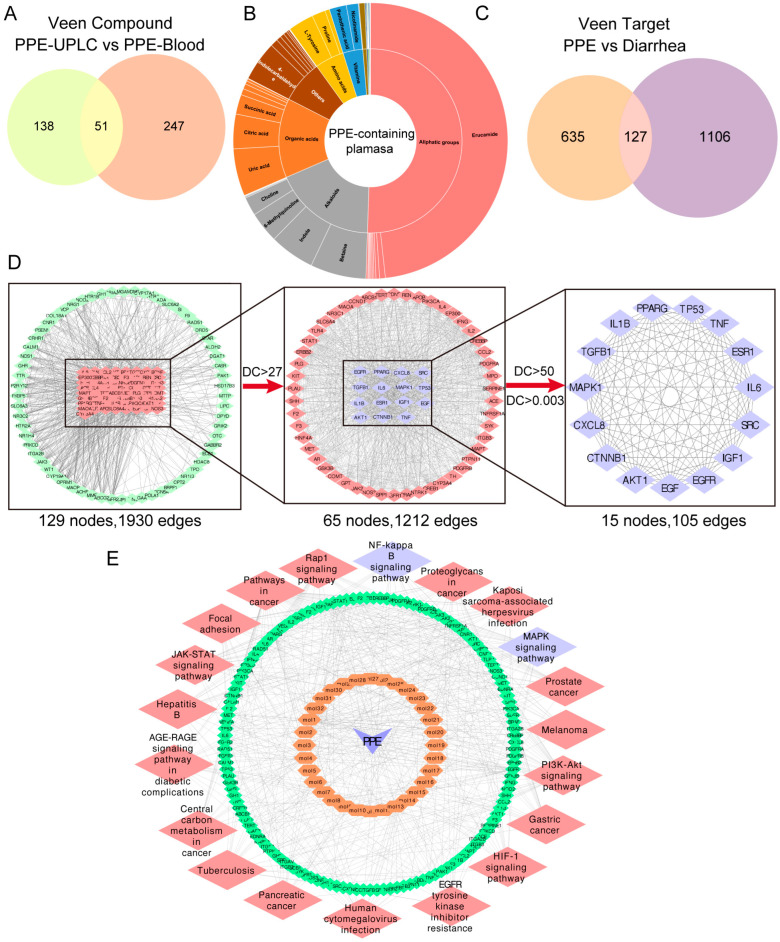
Network pharmacology combined with serum pharmacochemistry for mechanistic analysis of PPE on IBS-D. (**A**) Intersection of mass spectrometry components of PPE with components identified in serum. (**B**) Classification of prototype components that enter the bloodstream. (**C**) Overlap between the targets of mass spectrometry components and blood-entry components. (**D**) Identification of core targets within the protein–protein interaction (PPI) network. (**E**) KEGG enrichment to analyze potential signaling pathways.

To identify potential bioactive components of PPE responsible for its therapeutic effect, we conducted a comprehensive analysis of the chemical composition of plasma from both healthy rats and rats administered PPE (Figure S1). A total of 298 compounds were identified, and 51 plasma components were specifically selected for further investigation compared to blank plasma (Figure 4A). These components included fatty acids (15, 24.59%), organic acids (9, 14.75%), alkaloids (8, 13.11%), amino acids (4, 6.55%), vitamins (3, 4.92%), phenylpropanes (3, 4.92%), phenolics (2, 3.28%), saccharides (1, 1.64%), terpenoids (1, 1.64%), nucleosides (1, 1.64%), lignans (1, 1.64%), flavonoids (1, 1.64%), steroid analogs (1, 1.64%), and others (11, 18.03%) (Figure 4B). From these identified components, 762 corresponding targets were obtained via an open-access database of bioactive compounds. Simultaneously, 1233 disease-related targets were retrieved from multiple disease databases. By comparing the two datasets, 127 possible therapeutic targets were identified (Figure 4C). The protein–protein interaction (PPI) network, which consisted of 129 nodes and 1930 edges, was constructed through the STRING platform. A total of 15 core targets and 105 edges were selected based on Degree Centrality (DC) and Betweenness Centrality (BC) (Figure 4D). Finally, pathway enrichment analysis through the DAVID database showed significant involvement of the NF-κB, MAPK, Rap1, PI3K-Akt, HIF-1, and JAK-STAT signaling pathways (Figure 4E). Collectively, these findings suggest that PPE exerts its therapeutic effects in IBS-D through the modulation of key signaling pathways, particularly NF-κB and MAPK.

### 3.5. NF-κB and MAPK Pathways Are Key Mechanisms for IBS-D Alleviation

**Figure 5:**
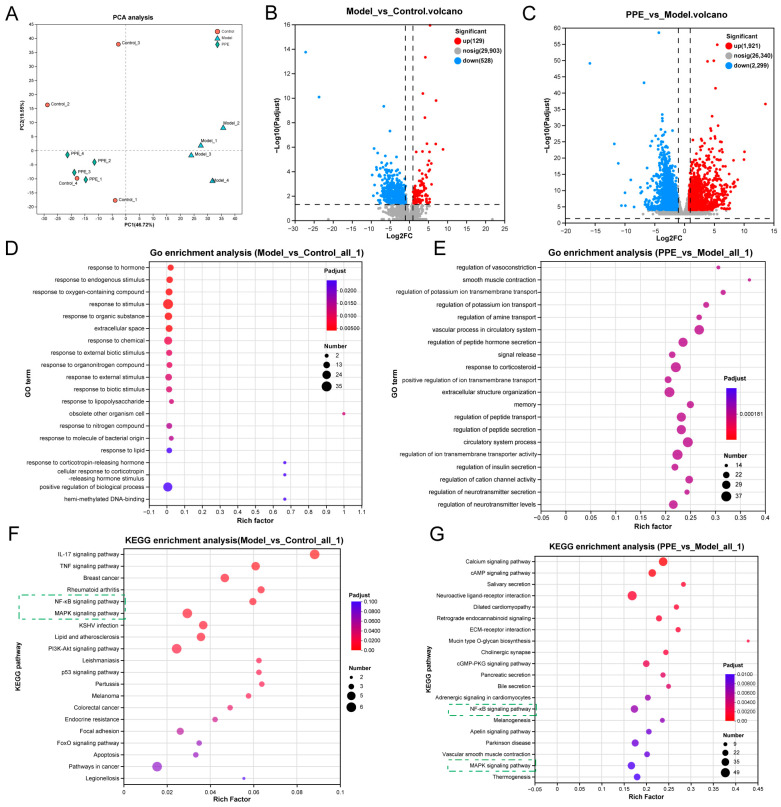
Transcriptomic analysis of PPE on diarrhea in IBS-D. (**A**) Results of principal component analysis (PCA). (**B**) Volcano plot comparing Model and Control groups. (**C**) Volcano plot comparing PPE and Model groups. (**D**) GO enrichment analysis between Model and Control groups. (**E**) GO enrichment analysis between PPE and Model groups. (**F**) KEGG enrichment analysis comparing Model and Control groups. (**G**) KEGG enrichment analysis of PPE compared to the Model group.

To identify the signaling pathways involved in PPE’s alleviation of IBS-D, a transcriptomic analysis was conducted. The results indicated notable differences in gene expression among the PPE-treated, model, and control groups based on principal component analysis (PCA) (Figure 5A). Compared to the control group, the model group exhibited 129 up-regulated and 528 down-regulated genes (Figure 5B). In contrast, the PPE group demonstrated a substantial increase, with 1821 genes up-regulated and 2299 genes down-regulated relative to the model group (Figure 5C). Additionally, Gene Ontology (GO) enrichment analysis revealed that biological processes, such as hormonal responses, signal transduction, and regulation of transit peptides, were enriched across the three groups (Figure 5D,E). Kyoto Encyclopedia of Genes and Genomes (KEGG) enrichment analysis further identified the MAPK and NF-κB signaling pathways as key mechanisms through which PPE regulates IBS-D (Figure 5F,G). Moreover, analysis of differential gene expression within the MAPK and NF-κB pathways demonstrated that PPE treatment significantly down-regulated key genes, including Mapk10, Mapk8ip2, Gadd45g, Fos, Jun, and Map3k8 in the MAPK pathway (Figure S2A,B). Similarly, within the NF-κB pathway, Cxcl2, Cxcl1, Ptgs2, Gadd45g, and Jun were consistently down-regulated following PPE treatment (Figure S2C,D). These results revealed that PPE significantly alters gene expression in IBS-D, identifying the MAPK and NF-κB signaling pathways as key mechanisms underlying its therapeutic effects.

### 3.6. Molecular Docking Results

**Figure 6:**
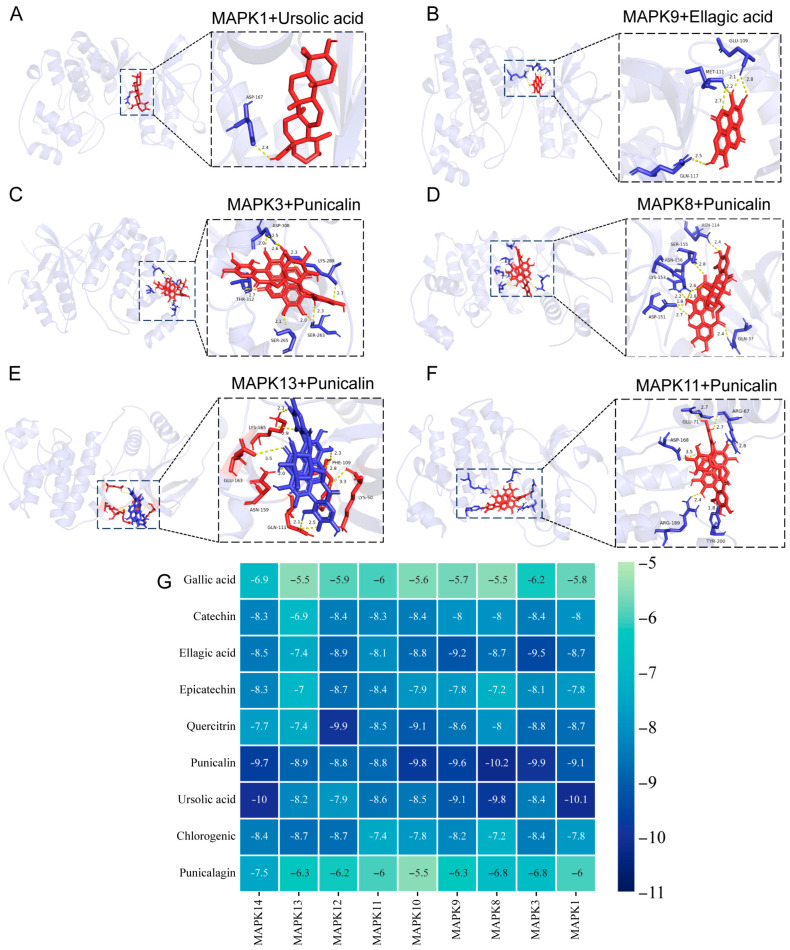
Molecular docking of major components in PPE with key targets in the MAPK signaling pathway. (**A**) Molecular docking of MAPK1 with Ursolic acid. (**B**) Molecular docking of MAPK9 with Ellagic acid. (**C**) Molecular docking of MAPK3 with Punicalin. (**D**) Molecular docking of MAPK8 with Punicalin. (**E**) Molecular docking of MAPK13 with Punicalin. (**F**) Molecular docking of MAPK11 with Punicalin. (**G**) Post-docking binding energy thermograms for nine components of PPE with nine major proteins in the MAPK signaling pathway.

**Figure 7:**
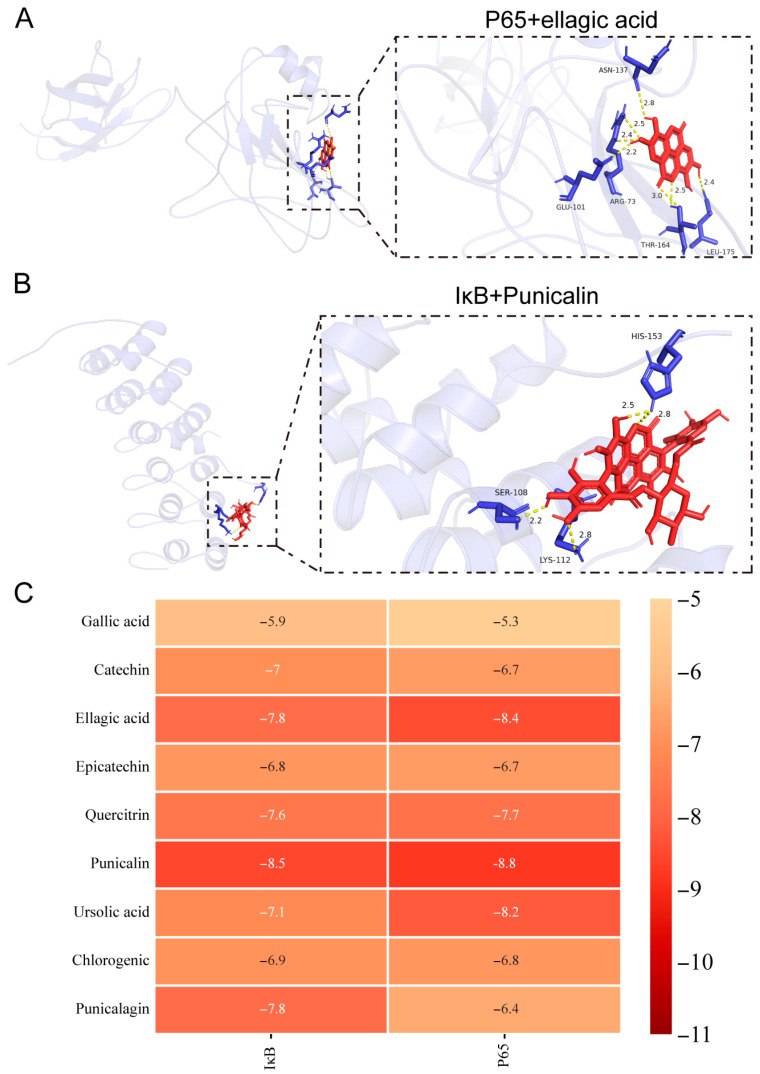
Molecular docking of major components in PPE with key targets in the NF-κB signaling pathway. (**A**) Molecular docking of P65 with Ellagicacid. (**B**) Molecular docking of IκB with Punicalin. (**C**) Binding energy thermograms for nine components of PPE following docking with P65 and IκB proteins.

To elucidate the role of PPE in the NF-κB and MAPK signaling pathways, we selected nine major bioactive components for molecular docking to investigate the interactions of nine major active components of PPE with these pathways according to the reported results. The docking scores for all interactions were below −5 kcal/mol, indicating high affinity (Figure 6A–F). Notably, the binding energies of ellagic acid, epicatechin, quercitrin, punicalin, and chlorogenic acid with all nine MAPK proteins were below −7 kcal/mol (Figure 6G). Furthermore, we performed docking of the nine components with the P65 and IκB proteins within the NF-κB signaling pathway, which also yielded docking scores under −5 kcal/mol, signifying a strong affinity for both P65 and IκB proteins. Additionally, the binding energies of ellagic acid, quercitrin, punicalin, and ursolic acid with the two proteins were all below −7 kcal/mol (Figure 7A–C). These findings suggest that the primary components of PPE can effectively interact with the MAPK and NF-κB signaling pathways, thereby providing a theoretical basis for the use of PPE in the treatment of IBS-D.

### 3.7. Inhibition of MAPK and NF-κB Signaling Pathways by PPE in IBS-D Rats

**Figure 8:**
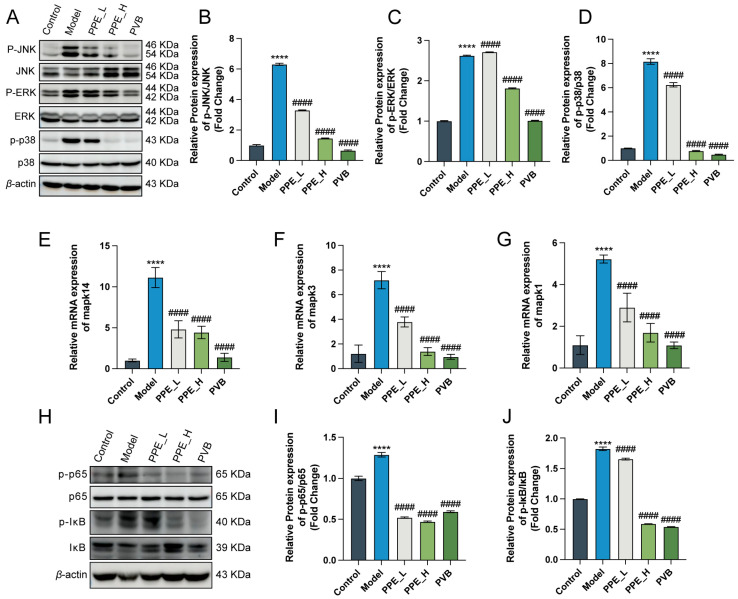
PPE inhibits MAPK and NF-κB signaling pathways in IBS-D rats. (**A**) Expression levels of major proteins in the MAPK signaling pathway in IBS-D after PPE administration. (**B**–**D**) Quantitative analysis of the expression levels of p-JNK, p-ERK, p-P38 in the MAPK signaling pathway following PPE administration. n = 3. (**E**–**G**) The mRNA levels of mapk14, mapk3, mapk1. n = 6. (**H**) Expression levels of major proteins in the NF-κB signaling pathway in IBS-D after PPE administration. (**I**,**J**) Quantitative analysis of expression levels of p-P65, p-IκB in the NF-κB signaling pathway in IBS-D after PPE. n = 3. **** *p* < 0.0001 compared to the control group, #### *p* < 0.0001 compared to the model group.

To further validate the findings from our network pharmacology and molecular docking studies, we performed WB assays to investigate the activation of the MAPK and NF-κB signaling pathways. The results revealed a significant up-regulation of phosphorylated JNK (P-JNK), ERK (P-ERK), and P38 (P-P38) in the IBS-D rat model, indicating a state of oxidative stress. Notably, treatment with PPE reversed this trend, leading to a significant reduction in the expression of these proteins (Figure 8A–D). Furthermore, we analyzed the mRNA levels of key proteins within the MAPK signaling pathways, confirming that their expression was significantly elevated in the model group and significantly decreased following PPE administration (Figure 8E–G). NF-κB signaling pathway demonstrated a similar trend (Figure 8H–J). Collectively, these findings suggested that PPE alleviates symptoms, inflammatory responses, and oxidative stress in IBS-D rats by inhibiting the MAPK and NF-κB signaling pathways.

## 4. Discussion

IBS-D is a chronic functional gastrointestinal disorder characterized by clinical symptoms such as diarrhea, abdominal pain, abdominal distension, and recurrent episodes. This condition significantly diminishes the patient’s quality of life and imposes a heavy burden on both the family and society [36]. Pomegranate peel refers to the pericarp of the pomegranate (*Punica granatum*), a member of the family *Punicaceae*. Research has shown that PPE modulates the imbalance of intestinal flora induced by a high-fat diet, which also alleviates colonic tissue damage and reduces intestinal inflammation [37].

The integrity of intestinal tight junctions is crucially linked to the pathophysiology of IBS-D [38]. An increase in the number of goblet cells up-regulates mucin expression, facilitating the repair of damage to the intestinal mucosal barrier [39]. In our study, we observed that PPE administration significantly elevated the number of goblet cells in IBS-D rats and enhanced the density of microvilli. This resulted in a more organized arrangement of microvilli on the intestinal surface and a reduction in the tight junction gap. The mucus layer, epithelial cells, and tight junction proteins are the primary components of the intestinal mucosal barrier [40,41]. These findings align with our results, where we found significantly lower levels of Claudin1, Occludin, and ZO-1, and higher levels of Claudin2 in the IBS-D group. PPE administration effectively reversed these trends, suggesting that PPE modulates the expression of intestinal barrier proteins and improves intestinal permeability. A reduction in the number of goblet cells can lead to a thinning of the mucosal layer, which in turn promotes intestinal inflammation [42].

The inflammatory response and oxidative stress are among the major factors contributing to the pathogenesis of intestinal diseases. Recent studies have shown that paeoniflorin significantly inhibits the levels of inflammatory factors (MPO, IL-6, IL-1β, and IL-10), thereby alleviating the inflammatory response in the intestinal mucosa of IBS-D [43]. Furthermore, several studies have shown that pomegranate possesses significant anti-inflammatory effects [44,45,46], which aligns with our findings. In our study, we observed that the levels of pro-inflammatory factors MPO, IL-6, and IL-1β in serum and intestinal tissues were significantly elevated, while the levels of the anti-inflammatory factor IL-10 were significantly reduced in the IBS-D group. Importantly, PPE reversed the trend of these indices, suggesting that it significantly alleviated the inflammatory response in IBS-D rats.

Oxidative stress is strongly associated with the intestinal inflammatory response in IBS-D [47,48,49,50]. These studies highlight the significance of inhibiting oxidative stress as a crucial strategy in the treatment of IBS-D. Additionally, PPE has been shown to increase glutathione levels, decrease MDA and nitric oxide levels, and restore dopamine, serotonin, catalase, and acetylcholinesterase levels to baseline levels after titanium poisoning [51]. Similarly, our results demonstrated that the levels of MDA, T-AOC, GSH-Px, and CAT were all reversed and returned to normal levels following PPE administration. Collectively, these results suggested that PPE significantly alleviated the inflammatory response and enhanced the antioxidant capacity of IBS-D.

Network pharmacology, in combination with serum medicinal chemistry, joint molecular docking, and transcriptomics, offers an integrated approach to studying the blood components and mechanisms of disease at a deeper level [52]. This strategy aligns with the characteristics of traditional Chinese medicine, which is known for its multiple pathways, multi-target, and multi-component approach [53]. Our previous study utilized this strategy to elucidate the potential mechanism of Baizhu Shaoyao decoction in alleviating IBS-D through the FOXO signaling pathway [35]. In the current study, after screening the active components and their targets, KEGG enrichment analysis of the core targets revealed that the potential mechanism by which PPE alleviates IBS-D may involve the NF-KB signaling pathway, MAPK signaling pathway, PI3K-AKT signaling pathway, HIF-1 signaling pathway, and JAK-STAT signaling pathway. Transcriptomics typically explores the direct alterations in differential gene expression, which are enriched in specific biological processes or signaling pathways, thus helping to reveal possible underlying mechanisms [54]. The mechanism of treating periodontitis may be achieved by modulating the PI3K/AKT and NF-κB/MAPK signaling pathways, as demonstrated by Ermiao Wan through network pharmacology as well as molecular docking [55]. Similarly, this study employed transcriptomics and molecular docking to validate the results from network pharmacology and serum medicinal chemistry, demonstrating that PPE alleviates IBS-D by inhibiting the up-regulation of key proteins in the NF-κB and MAPK signaling pathways.

The NF-κB and MAPK signaling pathways are integral to the pathogenesis of a wide range of inflammatory diseases, making them potential therapeutic targets for inflammation [56]. In this context, the examination of the sigmoid colon in 14 IBS-D patients revealed alterations in the intestinal barrier and visceral sensitivity, which may be related to changes in neuronal activity as well as aberrations in the MAPK signaling pathway [57]. This connection has also been observed in the fecal supernatants of IBS-D patients, where brain-derived neurotrophic factor (BDNF) was predominantly colonic [21]. One study demonstrated that the use of WenTongGanPi Decoction (WTGPD) effectively alleviated IBS-D and improved the intestinal barrier by inhibiting the MAPK signaling pathway, while also ameliorating micro-ecological dysregulation [20]. Similarly, another study confirmed that amphioxus glycosides significantly alleviated pulmonary edema, with its mechanism of action possibly involving inhibition of LPS toxicity by targeting the NF-κB and MAPK signaling pathways [58]. Our results indicated that PPE significantly reduced the expression of p-p-JNK, p-p38, and p-Erk proteins, which was further confirmed by qRT-PCR analysis. In addition, ellagic acid and andrographolide have been shown to ameliorate pathological lung injury and the inflammatory response by blocking the degradation and phosphorylation of IκBα, thereby preventing the activation of nuclear factor-κB (NF-κB) [45]. Our findings were consistent with these studies, demonstrating that PPE significantly reduced the expression of p-p65, p-IκB, and the ratio of p-p65/p65, as well as the ratio of p-IκB/P-IκB. These results suggested that PPE may mitigate IBS-D symptoms by inhibiting the activation of both the MAPK and NF-κB signaling pathways.

In summary, PPE alleviates the symptoms of IBS-D, including diarrhea, by mitigating the inflammatory response, enhancing antioxidant capacity, and reducing intestinal damage. Additionally, it strengthens the protective effect of the intestinal barrier, which may occur through the inhibition of the MAPK and NF-κB signaling pathways, thereby exerting an anti-IBS-D effect. However, it is important to note that this study lacks the support of in vitro experiments, and we plan to conduct such experiments in the future to further validate this mechanism and fully analyze its potential. Moreover, this study provides a theoretical basis for exploring the potential of PPE in the treatment of IBS-D and offers valuable direction for our future research.

## 5. Conclusions

PPE alleviates IBS-D symptoms by down-regulating the MAPK and NF-κB signaling pathways, positioning it as a promising therapeutic agent for IBS-D treatment.

## Figures and Tables

**Table 1:** Gene-specific primer sequence for qRT-PCR.

Genes	Primer Forward (5′ to 3′)	Primer Reverse (5′ to 3′)	Production Length (bp)	Accession No.
Claudin-1	CTTCTGGGTTTCATCCTGGCTTCG	CCTGAGCAGTCACGATGTTGTCC	112	NM_031699.3
ZO-1	CCATCTTTGGACCGATTGCTG	TAATGCCCGAGCTCCGATG	123	NM_001106266.1
Claudin-2	CGCAACTCAGAGACCGCTAACG	ATGGGAGAGAAAGGGCTGGAGATC	129	NM_001308255.1
Occludin	GTCTTGGGAGCCTTGACATCTTG	GCATTGGTCGAACGTGCATC	174	NM_031329.3
MAPK14/p38	AAGACTTCCCAGCAGTCCTATC	CTGGAGGATCAGTTGTGTTCAA	103	NM_031020.3
MAPK3	GTAGACGGTTCTGGAATGGAAG	AGGAATGTTCTGTCAGGGAAAA	179	NM_017347.3
MAPK1	CGCTACACTAATCTCTCGTACA	GCAACTCGAACTTTGTTGAGAT	86	NM_053842.2
GAPDH	GCAAGTTCAACGGCACAG	GCCAGTAGACTCCACGACAT	140	NM_017008.4

**Table 2:** The main potential active compounds of PPE.

Name	Formula	Annot. Delta Mass [ppm]	Calc. MW	*m*/*z*	RT [min]	Reference Ion
Ellagic acid	C_14_H_6_O_8_	−6.69	302.00425	300.99701	24.815	[M − H]^−1^
Gallic acid	C_7_H_6_O_5_	−6.61	170.0204	169.01312	7.879	[M − H]^−1^
Quercitrin	C_21_H_20_O_11_	−7.6	448.09715	449.10443	23.79	[M + H]^+1^
Punicalin	C_34_H_22_O_22_	−7.7	782.05425	783.06152	22.252	[M + H]^+1^
Ursolic acid	C_30_H_48_O_3_	−6.83	456.35723	457.36438	48.132	[M + H]^+1^
Epicatechin	C_15_H_14_O_6_	−7.33	290.07691	291.08401	22.721	[M + H]^+1^

## Data Availability

All data that support the findings of this study are available from the corresponding author upon request. They are not publicly available due to privacy reasons and because they are part of ongoing study.

## Supplementary Materials

The following supporting information can be downloaded at: https://www.mdpi.com/article/10.3390/nu16223854/s1, Figure S1: Major chemical constituents in plasma after PPE administration; Figure S2: Regulation effect of major differential genes in the MAPK and NF-κB signaling pathways after PPE administration; Table S1: Detailed information on the mobile phase; Table S2: Bristol stool scores; Table S3: The specific information on the main compounds of PPE.
