# Supplementary material for: Pomegranate Peel Extract Mitigates Diarrhea-Predominant Irritable Bowel Syndromes via MAPK and NF-κB Pathway Modulation in Rats
Source: Nutrients. 2024 Nov 11;16(22):3854. doi: 10.3390/nu16223854 (PMC11597445; doi:10.3390/nu16223854)
Supplement: Supplementary file 1 [file nutrients-16-03854-s001.zip › nutrients-3278256-supplementary.pdf]

Supplementary information for Original Article

S1 Materials and methods

S1.1 Main chemical information of PPE by UHPLC-MS/MS

To identify the main components of PPE, this study was a UHPLC-MS/MS system performed with Q Exactive Orbitrap (Thermo Fisher Scientific, USA), a high-resolution tandem mass spectrometer with an iron source. The chromatographic separation was performed on an ACQUITY UPLC HSS T3 column (2.1 × 100 mm, 1.8 μm, Waters) at a flow rate of 0.3 ml/min with the column temperature kept at 35 °C. The mass spectral analysis was obtained in full MS-ddMS^2 mode and full scan analysis. The parameters are as follows: scan range, m/z 100-1200; MS^1 and MS^2 resolution, 70,000 and 17,500. The data were performed in both positive and negative ionization modes for qualitative analysis of the samples. Compound Discoverer 3.3 software was employed to acquire raw data, and both the local and online MzCloud databases were simultaneously utilized to identify compounds.

Table S1 Detailed information on the mobile phase.

Time	Flow Rate（mL/min）	%A	%B
0	0.3	100	0
10	0.3	70	30
25	0.3	60	40
30	0.3	50	50
40	0.3	30	70
45	0.3	0	100
60	0.3	0	100
60.5	0.3	100	0
70	0.3	100	0

S1.2 The details of Abdominal withdrawal reflex (AWR)

A balloon coated with petroleum jelly was inserted into the distal colon about 8 cm from the anus and colorectal dilatation was maintained by water injection. AWR scores were following standard: 0 point: no behavioral response; 1 point: sample head movement followed by immobility; 2 points: contraction of abdominal muscles; 3 points: lifting the abdomen; 4 points: arching of the body and lifting of pelvic structures.

S1.3 Bristol stool scores

Table S2 Bristol stool scores.

Score	Character
1.Pyreniform	Hard and lumpy, like a sheep’s stool

2.Dry hard shape	The texture is hard, the surface is convex
3.There are folds	Banana-shaped, with a wrinkled surface
4. Banana-shape	Banana-shaped, smooth surface
5.Soft poop	The texture is soft and semi-solid
6.Porridge-like	No fixed shape, atherosclerosis
7.Water like	No solids, no water samples

S1.4 Plasma sample processing and mass spectrometry conditions

Plasma samples and PPE were treated with methanol prior to analysis using a UHPLC-Q-Orbitrap system. Specifically, protein precipitation was achieved by mixing 200 μL of serum with 800 μL of a 1:1 acetonitrile-methanol solution. The mixture was vortexed for 30 seconds and subsequently sonicated for 10 minutes. Following sonication, the mixture was centrifuged at 14,000 rpm and 4 °C for 10 minutes. Subsequently, 800 μL of the supernatant was transferred to a centrifuge tube, subjected to a second centrifugation, and the resulting supernatant was collected in an injection vial for UHPLC-MS/MS analysis.

Chromatographic separation was performed using a Waters ACQUITY UPLC HSS T3 C18 column (2.1 mm × 100 mm, 1.8 μm; Waters Corporation, USA), with the column temperature maintained at 35 °C and a flow rate of 0.2 mL/min. The mobile phase consisted of 0.1% formic acid in acetonitrile (A) and 0.1% formic acid in water (B). Both positive and negative ion modes of the mass spectrometer were employed for analysis, with ion discharge voltages set at 3.2 kV for positive mode and 3.0 kV for negative mode. The mass scan range for positive ion mode was set from 100 to 1500 m/z. Data analysis was conducted using Compound Discoverer 3.3, along with mzCloud and mzVault databases.

S2 Results

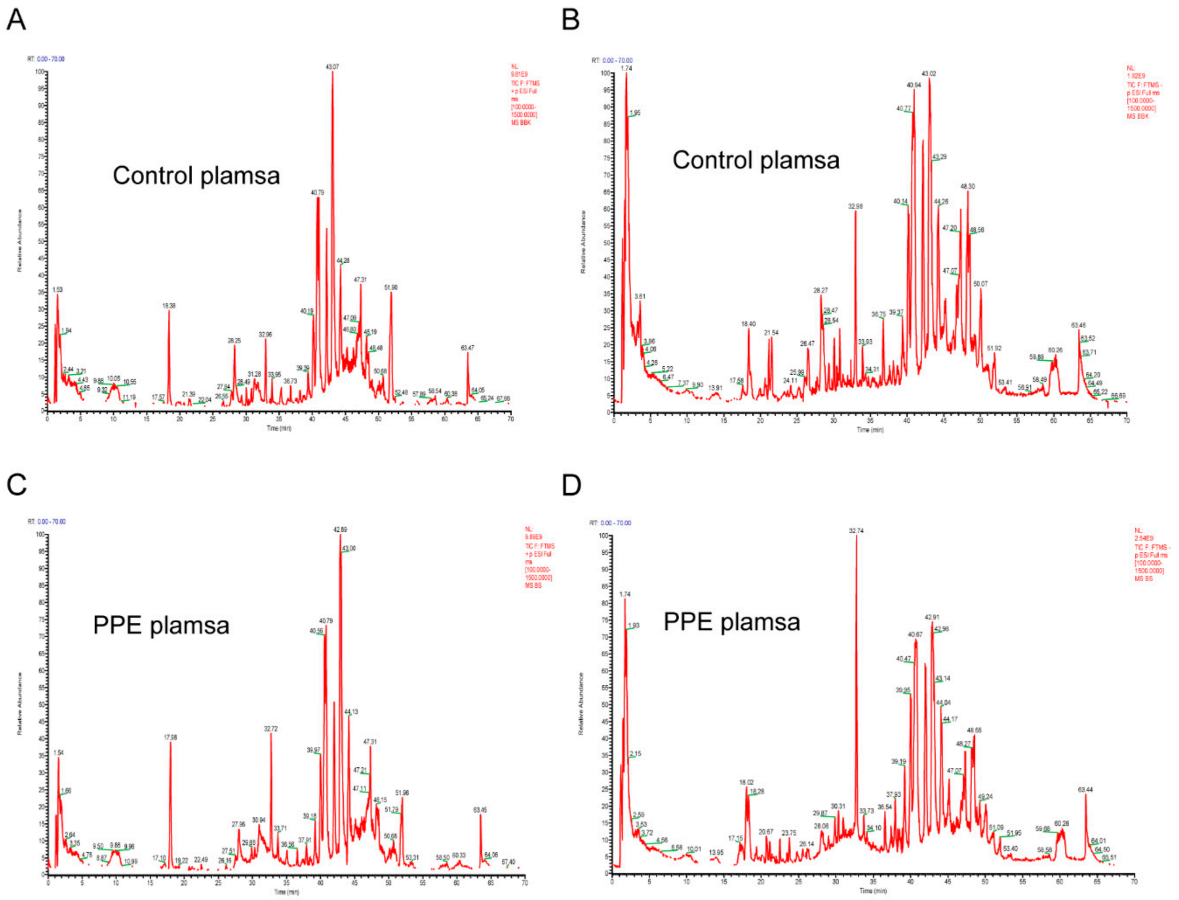

Figure S1 Major chemical constituents in plasma after PPE administration. (A and B) Total ion flow plots in positive and negative modes in control plasma. (C and D) Total ion flow plots in positive and negative modes in PPE administered plasma.

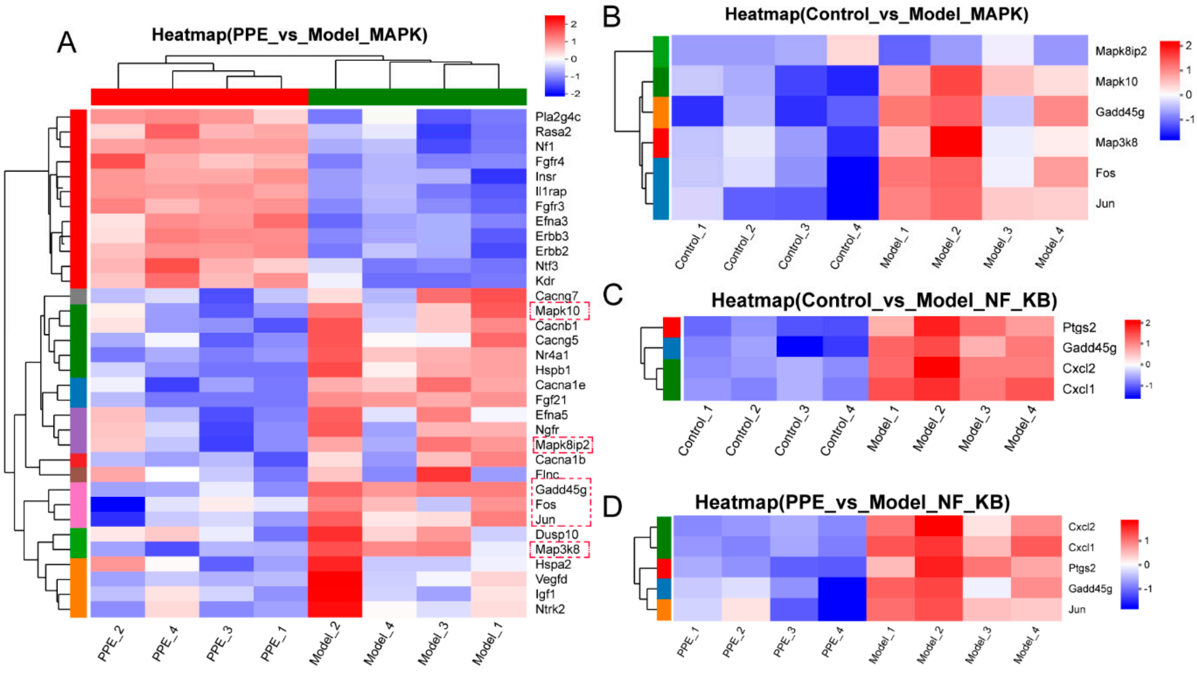

Figure S2 Regulation effect of major differential genes in the MAPK and NF-κB signaling pathway after PPE administration. (A and B) Differential genes in MAPK signaling pathway

after PPE-treated. (C and D) Differential genes in NF-κB signaling pathway after PPE-treated.

Table S3 The specific information on the main compounds of PPE.

Name	Formula	Annot. DeltaMass [ppm]	Calc. MW	m/z	RT [min]	Reference Ion	Group Area	Categorisation
Ellagic acid	C14 H6 O8	-6.69	302.00425	300.99701	24.815	[M-H]-1	15036562665	phenols
Corilagin	C27 H22 O18	-6.36	634.07658	633.06934	23.054	[M-H]-1	5505117799	phenols
Schisandrin B	C23 H28 O6	-7.56	400.18556	401.19287	46.416	[M+H]+1	5421061956	lignans
Citric acid	C6 H8 O7	-6.59	192.02574	191.01849	3.689	[M-H]-1	4567322792	organic acids
Gluconic acid	C6 H12 O7	-7.09	196.05691	195.04964	1.622	[M-H]-1	3251178625	organic acids
Schizandrin A	C24 H32 O6	-7.88	416.21661	417.22391	45.616	[M+H]+1	2825967603	lignans
Oleamide	C18 H35 N O	-7.48	281.26976	282.27704	48.353	[M+H]+1	2137439002	amides
L-Pyroglutamic acid	C5 H7 N O3	-7.11	129.04168	130.04889	3.928	[M+H]+1	1994449607	amino acids
Choline	C5 H13 N O	-8.62	103.09883	104.1061	1.577	[M+H]+1	1762519565	amino alcohols
Methyl gallate	C8 H8 O5	-6.68	184.03594	183.02867	22.717	[M-H]-1	1655504354	amino acids
Trigonelline	C7 H7 N O2	-7.41	137.04666	138.05394	1.751	[M+H]+1	1563556185	alkaloids
Geraniin	C41 H28 O27	-6.44	952.07566	951.06848	23.773	[M-H]-1	1391990925	phenols
Gallic acid	C7 H6 O5	-6.61	170.0204	169.01312	7.879	[M-H]-1	1278396900	phenols
Pyrogallol	C6 H6 O3	-7.04	126.03081	125.02354	8.125	[M-H]-1	1153717129	phenols
L-(-)-Malic acid	C4 H6 O5	-6.79	134.02061	133.01334	1.87	[M-H]-1	1015728167	organic acids
Schisandrin	C24 H32 O7	-8.04	432.21133	433.21866	36.626	[M+H]+1	845056925.7	lignans
Asiatic acid	C30 H48 O5	-6.88	488.34681	487.33966	37.037	[M-H]-1	770073713.3	terpenoids
1,6-Bis-O-(3,4,5-trihydroxybenzoyl)hexopyranose	C20 H20 O14	-6.73	484.08204	483.07477	22.232	[M-H]-1	756323980.5	glycosides
L-Threonic acid	C4 H8 O5	-6.54	136.03628	135.02901	1.686	[M-H]-1	630944628.8	organic acids
Valpromide	C8 H17 N O	-7.57	143.12993	144.13721	10.006	[M+H]+1	592193956.6	amides
Betaine	C5 H11 N O2	-8.25	117.07801	118.08529	1.658	[M+H]+1	575423868.1	others
β-D-Glucopyranuronic acid	C6 H10 O7	-6.79	194.04133	193.03406	1.618	[M-H]-1	520875559.7	glycosides
Punicalin	C34 H22 O22	-7.7	782.05425	783.06152	22.252	[M+H]+1	500775253.3	flavonoids
α,α-Trehalose	C12 H22 O11	-8.36	342.11335	381.07629	1.681	[M+K]+1	450969421.7	others
(15Z)-9,12,13-Trihydroxy-15-octadecenoic acid	C18 H34 O5	-6.67	330.23842	329.23115	31.906	[M-H]-1	443916508	fatty acids
Hexadecanamide	C16 H33 N O	-6.66	255.25451	256.26179	47.948	[M+H]+1	417551127.7	amides
2-Furoic acid	C5 H4 O3	-7.16	112.01524	111.00797	3.645	[M-H]-1	370800106	organic acids

D-(-)-Mannitol	C6 H14 O6	-7.73	182.07763	181.07063	1.604	[M-H]-1	369758980.6	others
Epigallocatechin	C15 H14 O7	-7.17	306.07176	307.07877	21.312	[M+H]+1	342414315.4	flavonoids
Gomisin J	C22 H28 O6	-7.79	388.18556	389.19284	37.789	[M+H]+1	325574423.4	lignans
Schisanhenol	C23 H30 O6	-7.55	402.2012	403.2085	42.255	[M+H]+1	307213637.9	lignans
Erucamide	C22 H43 N O	-7.61	337.3319	338.33917	52.349	[M+H]+1	302053060.6	amides
D-(+)-Galactose	C6 H12 O6	-6.55	180.06221	179.05493	1.703	[M-H]-1	232087684.6	glycosides
Ursonic acid	C30 H46 O3	-7.35	454.34136	455.34863	44.366	[M+H]+1	227800138	terpenoids
5-Hydroxymethyl-2-furaldehyde	C6 H6 O3	-7.81	126.03071	127.03799	1.639	[M+H]+1	219932866.5	others
16-Hydroxyhexadecanoic acid	C16 H32 O3	-6.25	272.23344	271.22617	47.277	[M-H]-1	217854839.4	fatty acids
Kojic acid	C6 H6 O4	-7.17	142.02559	143.03287	6.003	[M+H]+1	216834396.6	phenols
L-Glutamic acid	C5 H9 N O4	-7.78	147.05201	295.1113	1.596	[2M+H]+1	216813984.7	amino acids
trans-3-Indoleacrylic acid	C11 H9 N O2	-7.6	187.06191	188.06918	21.859	[M+H]+1	211621918.7	others
Tropine	C8 H15 N O	-7.29	141.11434	142.12161	1.856	[M+H]+1	200099350.8	alkaloids
8-Hydroxyquinoline	C9 H7 N O	-7.76	145.05164	146.05891	21.169	[M+H]+1	199911344.5	others
3-Hydroxy-3-(methoxycarbonyl)pentanedioic acid	C7 H10 O7	-6.4	206.04133	205.03406	12.954	[M-H]-1	197523157.7	organic acids
Adenine	C5 H5 N5	-7.11	135.05354	136.06081	2.96	[M+H]+1	177851461.4	others
Dihydrozeatin	C10 H15 N5 O	-7.19	221.12607	222.13335	21.517	[M+H]+1	173072444.3	others
Nicotinamide	C6 H6 N2 O	-8.24	122.04701	123.05428	3.471	[M+H]+1	162272087.7	vitamins
1,2,3,6-Tetra-O-galloyl-β-D-glucose	C34 H28 O22	-6.05	788.10246	787.09521	24.366	[M-H]-1	158596223.4	others
Fumaric acid	C4 H4 O4	-7.34	116.01011	115.00283	1.862	[M-H]-1	153948439.5	organic acids
Proline	C5 H9 N O2	-7.78	115.06243	116.06971	1.754	[M+H]+1	149327713.8	amino acids
18-β-Glycyrrhetinic acid	C30 H46 O4	-6.25	470.33667	469.3295	48.88	[M-H]-1	148679474	terpenoids
Astragalin	C21 H20 O11	-6.69	448.09756	447.09067	25.795	[M-H]-1	146899194	flavonoids
Cynaroside	C21 H20 O11	-7.67	448.09712	449.1044	26.052	[M+H]+1	145087139.6	flavonoids
Stearamide	C18 H37 N O	-6.93	283.28555	284.29283	50.134	[M+H]+1	140207093.3	amides
Isoquercitrin	C21 H20 O12	-6.77	464.09233	465.09943	24.952	[M+H]+1	138643239	flavonoids
Corchorifatty acid F	C18 H32 O5	-6.28	328.22291	327.21564	30.777	[M-H]-1	138219069	fatty acids
Ursolic acid	C30 H48 O3	-6.83	456.35723	457.36438	48.132	[M+H]+1	137338736.2	terpenoids
Lorazepam	C15 H10 Cl2 N2 O2	7.39	320.0143	321.02158	1.756	[M+H]+1	136680059.8	others
L-Tryptophan	C11 H12 N2 O2	-7.63	204.08832	205.0956	21.858	[M+H]+1	132776193.4	amino acids
α-Cyperone	C15 H22 O	-7.33	218.16547	437.33823	44.131	[2M+H]+1	131097455	terpenoids
Robinetin	C15 H10 O7	-9.1	302.0399	303.04718	24.954	[M+H]+1	128243650.2	flavonoids

Kaempferol	C15 H10 O6	-7.97	286.04546	287.05273	25.796	[M+H]+1	127112065.2	flavonoids
Phytosphingosine	C18 H39 N O3	-7.53	317.29061	318.29788	37.896	[M+H]+1	120916009.2	amino alcohols
Ethyl gallate	C9 H10 O5	-6.24	198.05159	197.04431	24.694	[M-H]-1	120326516.1	others
Oleanonic acid	C30 H46 O3	-7.35	454.34136	455.34863	44.13	[M+H]+1	119549715.9	terpenoids
Epicatechin	C15 H14 O6	-7.33	290.07691	291.08401	22.721	[M+H]+1	117248084.3	flavonoids
Lactitol	C12 H24 O11	-7.02	344.12944	343.12216	1.747	[M-H]-1	116516009.2	glycosides
3-[(Carboxycarbonyl)amino]-L-alanine	C5 H8 N2 O5	-461.4	175.9621	174.95482	1.307	[M-H]-1	115810450.6	others
Itaconic acid	C5 H6 O4	-6.68	130.02574	129.01846	1.734	[M-H]-1	114928830.9	organic acids
D-Glucosamine	C6 H13 N O5	-7.81	179.07797	180.08525	1.644	[M+H]+1	108397973.8	glycosides
Apigenin-7-O-β-D-glucoside	C21 H20 O10	-7.13	432.10257	433.10965	26.204	[M+H]+1	107212910.4	flavonoids
4-Hydroxybenzoic acid	C7 H6 O3	-7.63	138.03064	139.03792	21.311	[M+H]+1	99554560.29	others
L-Phenylalanine	C9 H11 N O2	-7.29	165.07778	166.08505	13.042	[M+H]+1	94343836.64	amino acids
L-Norleucine	C6 H13 N O2	-7.39	131.09366	132.10094	5.203	[M+H]+1	91050958.6	amino acids
N-Ethylglycine	C4 H9 N O2	-7.21	103.06259	104.06977	1.63	[M+H]+1	85095305.35	amino acids
Sorbic acid	C6 H8 O2	-7.82	112.05155	113.05883	21.685	[M+H]+1	81130283.1	organic acids
Epigallocatechin gallate	C22 H18 O11	-6.13	458.0821	457.07513	23.558	[M-H]-1	80891239.48	flavonoids
Kaempferol-3-O-rutinoside	C27 H30 O15	-6.2	594.15479	593.14801	25.269	[M-H]-1	80185552.01	flavonoids
δ-Gluconic acid δ-lactone	C6 H10 O6	-6.72	178.04654	355.08582	1.741	[2M-H]-1	78795872.87	others
Arjungenin	C30 H48 O6	-5.81	504.34216	503.3349	32.852	[M-H]-1	75964706.17	flavonoids
Uric acid	C5 H4 N4 O3	-7.24	168.02712	167.01991	3.92	[M-H]-1	75343360.39	others
DL-Glutamine	C5 H10 N2 O3	-8.06	146.06796	147.07524	1.571	[M+H]+1	71470616.06	amino acids
Glutaric anhydride	C5 H6 O3	-7.6	114.03083	113.02363	1.668	[M-H]-1	71274064.6	others
Quinic acid	C7 H12 O6	-6.54	192.06213	191.05486	1.753	[M-H]-1	70614758.09	organic acids
Succinic acid	C4 H6 O4	-6.52	118.02584	117.01856	4.632	[M-H]-1	69760778.32	organic acids
D-Mannose 6-phosphate	C6 H13 O9 P	-7.44	260.02778	259.02051	1.575	[M-H]-1	65518877.11	glycosides
Luteolin	C15 H10 O6	-6.79	286.04579	285.03876	29.039	[M-H]-1	60716191.15	flavonoids
L-Tyrosine	C9 H11 N O3	-7.46	181.07254	182.07982	5.634	[M+H]+1	60690146.41	amino acids
Indole	C8 H7 N	0.68	234.11379	118.06417	21.861	[M+2H]+2	60491316.83	others
DL-Stachydrine	C7 H13 N O2	-7.3	143.09358	144.10086	4.018	[M+H]+1	58637522.44	others
L-Valine	C5 H11 N O2	-8.25	117.07801	118.08529	2.5	[M+H]+1	56826186.32	amino acids
6-Methylquinoline	C10 H9 N	-7.22	143.07247	144.07974	22.778	[M+H]+1	55622592.1	others
Hyperoside	C21 H20 O12	-6.57	464.09243	465.0994	26.664	[M+H]+1	55547328.95	flavonoids
Glabrolide	C30 H44 O4	-7.3	468.32054	469.32782	38.562	[M+H]+1	54994493.8	terpenoids

1-Linoleoyl glycerol	C21 H38 O4	-7.36	354.2744	355.28168	47.358	[M+H]+1	53397142.55	others
5-Hydroxymethylfurfural	C6 H6 O3	-7.81	126.03071	127.03799	21.089	[M+H]+1	52840879.3	others
Quercetin	C15 H10 O7	-7.22	302.04047	301.03345	29.127	[M-H]-1	52317140.06	flavonoids
3-Methoxybenzaldehyde	C8 H8 O2	-7.39	136.05142	137.0587	23.021	[M+H]+1	52138303.28	aromatic aldehydes
2-Hydroxycinnamic acid	C9 H8 O3	-6.22	164.04632	163.03905	21.368	[M-H]-1	50366847.84	aromatic acids
2,3,4,9-Tetrahydro-1H-β-carboline-3-carboxylic acid	C12 H12 N2 O2	-7.49	216.08826	217.09554	22.786	[M+H]+1	47304004.47	Alkaloids
7,8-Dihydroxy-4-methylcoumarin	C10 H8 O4	-6.24	192.04106	191.03378	21.368	[M-H]-1	46793456.7	others
Pedunculoside	C36 H58 O10	2.49	696.40437	695.39709	30.87	[M-H]-1	45830664.72	terpenoids
1,2,3,4,6-Pentagalloylglucose	C41 H32 O26	-6.3	940.11226	469.0488	24.931	[M-2H]-2	45348882.27	phenols
9-Oxo-10(E),12(E)-octadecadienoic acid	C18 H30 O3	-7.14	294.21739	295.22464	44.703	[M+H]+1	44624418.94	amides
Kynurenic acid	C10 H7 N O3	-9.46	189.04081	190.04836	22.287	[M+H]+1	43809912.44	others
9-Oxo-ODE	C18 H30 O3	-7.45	294.2173	295.22458	31.905	[M+H]+1	43533878.72	amides
Phloridzin	C21 H24 O10	-6.26	436.13422	435.12698	26.675	[M-H]-1	42875967.67	flavonoids
Gomisin D	C28 H34 O10	-6.98	530.2115	531.21887	37.491	[M+H]+1	40988254.29	lignans
Rutin	C27 H30 O16	-6.4	610.14948	609.14258	24.498	[M-H]-1	40919460.95	flavonoids
Pantothenic acid	C9 H17 N O5	-7.79	219.10897	220.11627	21.204	[M+H]+1	39239198.23	vitamins
Puerarin	C21 H20 O9	-7.99	416.10741	417.11469	23.074	[M+H]+1	38344161.02	flavonoids
o-Veratraldehyde	C9 H10 O3	-6.92	166.06185	167.06912	21.509	[M+H]+1	37395806.61	aromatic aldehydes
Citramalic acid	C5 H8 O5	-6.32	148.03624	147.02896	4.842	[M-H]-1	36418877.7	organic acids
Coumarin	C9 H6 O2	-7.65	146.03566	147.04294	23.057	[M+H]+1	36358982.79	esters
(-)-Fustin	C15 H12 O6	-7.79	288.06114	289.06842	23.517	[M+H]+1	36316479.67	others
4-Indolecarbaldehyde	C9 H7 N O	-7.76	145.05164	146.05891	21.861	[M+H]+1	35833343.38	others
trans-Aconitic acid	C6 H6 O6	-7.22	174.01518	173.0079	2.617	[M-H]-1	35260979.53	organic acids
α-Eleostearic acid	C18 H30 O2	-7.4	278.22252	279.2298	43.215	[M+H]+1	35248592.98	fatty acids
(-)-Epigallocatechin	C15 H14 O7	-8.05	306.07149	307.07877	22.282	[M+H]+1	32688073.79	flavonoids
3-Hydroxy-3-methylglutaric acid	C6 H10 O5	-6.4	162.05179	161.04451	6.453	[M-H]-1	32534607.62	organic acids
Vicenin III	C26 H28 O14	-6.68	564.14414	565.15125	23.639	[M+H]+1	32299013.56	flavonoids
Oleoyl ethanolamide	C20 H39 N O2	-7.03	325.29579	326.30307	47.558	[M+H]+1	30598227.07	amino alcohols
Wilforlide A	C30 H46 O3	-7.35	454.34136	455.34863	47.634	[M+H]+1	30125383.29	terpenoids
Naringenin	C15 H12 O5	-7.72	272.06637	273.07358	26.086	[M+H]+1	29977209.75	flavonoids
Trilobatin	C21 H24 O10	-6.28	436.13421	435.12698	27.485	[M-H]-1	29303461.95	flavonoids
Nicotinic acid	C6 H5 N O2	-7.24	123.03114	124.03841	3.019	[M+H]+1	28465657.86	vitamins
L-Glutathione oxidized	C20 H32 N6 O12 S2	-7.68	612.14726	307.08075	8.016	[M+2H]+2	27634525.5	amino acids

2-Isopropylmalic acid	C7 H12 O5	-6.64	176.0673	175.06003	22.417	[M-H]-1	27477604.7	Organic acids
Shikimic acid	C7 H10 O5	-6.92	174.05162	173.04434	2.486	[M-H]-1	27441716.25	aromatic acids
Myricetin	C15 H10 O8	-6.6	318.03547	317.02832	26.911	[M-H]-1	27391272.63	flavonoids
7-Methoxycoumarin	C10 H8 O3	-7.33	176.04605	177.05333	23.714	[M+H]+1	26880686.86	others
(+/-)13-HODE	C18 H32 O3	-6.67	296.23317	295.22589	43.21	[M-H]-1	26577010.03	fatty acids
Uridine	C9 H12 N2 O6	-6.65	244.06791	243.06065	5.894	[M-H]-1	26262003.38	nucleosides
5'-S-Methyl-5'-thioadenosine	C11 H15 N5 O3 S	-7.92	297.08721	298.09448	21.893	[M+H]+1	25570566.24	others
18 β-Glycyrrhetintic Acid	C30 H46 O4	-6.99	470.33632	471.34348	43.893	[M+H]+1	24999334.75	terpenoids
CMPF	C12 H16 O5	-6.82	240.09814	239.091	24.244	[M-H]-1	24992143.31	phenols
Apigenin	C15 H10 O5	-7	270.05093	271.05804	30.967	[M+H]+1	24570880.95	flavonoids
19-Nortestosterone	C18 H26 O2	-7.55	274.19121	275.19849	30.792	[M+H]+1	24288606.27	hormones
4-(4-Nitrobenzyl)pyridine	C12 H10 N2 O2	-7.23	214.07268	215.07996	22.829	[M+H]+1	23822432.04	others
Brevifolincarboxylic acid	C13 H8 O8	-7.8	292.01964	293.02692	21.382	[M+H]+1	22056625.68	phenols
4-Methoxybenzaldehyde	C8 H8 O2	-7.39	136.05142	137.0587	26.35	[M+H]+1	20833923.6	phenols
Genistein	C15 H10 O5	-7.61	270.05077	271.05804	23.507	[M+H]+1	20764549.78	flavonoids
9(Z),11(E),13(E)-Octadecatrienoic Acid methyl ester	C19 H32 O2	-7.24	292.23812	293.24539	49.784	[M+H]+1	20629568.71	others
Uridine monophosphate (UMP)	C9 H13 N2 O9 P	-6.48	324.03377	323.02649	2.673	[M-H]-1	20439687.1	nucleosides
2-Hydroxy-4-methoxybenzaldehyde	C8 H8 O3	-7.29	152.04624	153.05351	24.811	[M+H]+1	20328612.86	aromatic aldehydes
Victoria Pure Blue BO	C33 H39 N3	-6.94	477.31108	478.31842	43.861	[M+H]+1	20175912.73	others
Methyl 4-hydroxy-3-methoxycinnamate	C11 H12 O4	-7.16	208.07207	209.07935	26.875	[M+H]+1	20054051.77	esters
Pyraclostrobin	C19 H18 Cl N3 O4	-7.08	387.09584	388.10312	43.43	[M+H]+1	19493260.67	esters
Phloretin	C15 H14 O5	-7.91	274.08196	275.08923	26.676	[M+H]+1	18841697.62	flavonoids
Riboflavin	C17 H20 N4 O6	-7.61	376.13542	377.1427	23.27	[M+H]+1	18204740.52	vitamins
9S,13R-12-Oxophytodienoic acid	C18 H28 O3	-7.84	292.20156	293.20883	30.783	[M+H]+1	18122992.47	fatty acids
Flusilazole	C16 H15 F2 N3 Si	-7.37	315.09801	316.10529	39.375	[M+H]+1	17976180.59	others
Pinolenic acid ethyl ester	C20 H34 O2	1.35	323.28014	324.28741	45.992	[M+H]+1	17026146	esters
Sedanolide	C12 H18 O2	-7.16	194.12929	195.13657	31.916	[M+H]+1	16452729.21	others
Angeloylgomisin H	C28 H36 O8	-7.43	500.2373	501.24457	39.466	[M+H]+1	15319604.13	lignans
(+/-)9,10-dihydroxy-12Z-octadecenoic acid	C18 H34 O4	-6.88	314.24355	313.23636	39.105	[M-H]-1	15183758.44	fatty acids
D-(+)-Fucose	C6 H12 O5	-6.75	164.06737	163.06009	2.514	[M-H]-1	14418161.31	glycosides
Dihydrosphingosine	C18 H39 N O2	-7.69	301.29576	302.30304	39.866	[M+H]+1	13968796.27	others
Quercitrin	C21 H20 O11	-7.6	448.09715	449.10443	23.79	[M+H]+1	13272882.82	flavonoids
(+/-)12(13)-DiHOME	C18 H34 O4	-6.92	314.24353	313.23636	38.733	[M-H]-1	13266012.9	others

Astragalin	C21 H20 O11	-7.67	448.09712	449.1044	25.272	[M+H]+1	13138306.28	flavonoids
Schisandrin C	C22 H24 O6	-7.5	384.15441	385.16168	43.884	[M+H]+1	13048035.36	lignans
Gentisic acid	C7 H6 O4	-6.24	154.02565	153.01837	14.782	[M-H]-1	13032215.48	organic acids
Guanosine	C10 H13 N5 O5	-6.51	283.08983	282.08255	13.905	[M-H]-1	12696524.93	nucleosides
Difenoconazole	C19 H17 Cl2 N3 O3	-7.57	405.06163	406.06891	42.054	[M+H]+1	12542706.69	others
Adenosine diphosphate ribose	C15 H23 N5 O14 P2	-6.49	559.06804	558.06085	2.645	[M-H]-1	12334461.16	nucleosides
3-Methoxy-5,7,3',4'-tetrahydroxy-flavone	C16 H12 O7	-6.43	316.05627	317.0632	29.14	[M+H]+1	12316266.48	flavonoids
Abscisic acid	C15 H20 O4	-7.04	264.1343	263.12726	28.598	[M-H]-1	12296829.45	hormones
Ethylmalonic acid	C5 H8 O4	-6.89	132.04135	131.03407	14.394	[M-H]-1	10896435.63	organic acids
Iristectorigenin B	C17 H14 O7	-7.2	330.07157	331.07858	31.268	[M+H]+1	10781875.89	flavonoids
Pinolenic acid	C18 H30 O2	-7.4	278.22252	279.2298	45.605	[M+H]+1	10467201.37	fatty acids
Monoolein	C21 H40 O4	-7.25	356.29008	357.29733	48.66	[M+H]+1	9396033.027	others
Coniferylaldehyde	C10 H10 O3	-7.05	178.06174	179.06902	26.879	[M+H]+1	9276950.079	others
Asiatic acid	C30 H48 O5	-7.25	488.34664	489.35391	37.586	[M+H]+1	8084900.156	terpenoids
Methyl 4-hydroxycinnamate	C10 H10 O3	-6.37	178.06186	177.05464	29.934	[M-H]-1	7621923.277	esters
Diosmetin	C16 H12 O6	-7.14	300.06125	301.06836	31.408	[M+H]+1	7506287.429	flavonoids
Bis(2-ethylhexyl) phthalate	C24 H38 O4	-7.62	390.27404	391.2814	51.118	[M+H]+1	6709288.282	esters
3-Acetoxyurs-12-en-23-oic acid	C32 H50 O4	-6.95	498.36745	499.37473	49.145	[M+H]+1	6694195.012	others
α-Linolenic acid	C18 H30 O2	-7.4	278.22252	279.2298	38.729	[M+H]+1	6439994.836	fatty acids
Avicularin	C20 H18 O11	-7.09	434.08183	435.08911	23.911	[M+H]+1	6182704.805	flavonoids
Daidzein	C15 H10 O4	-7.63	254.05597	255.06325	28.326	[M+H]+1	5844488.094	flavonoids
Progesterone	C21 H30 O2	-6.94	314.2224	315.22968	49.444	[M+H]+1	5708463.462	hormones
2-Hydroxymyristic acid	C14 H28 O3	-6.7	244.20221	243.19493	44.709	[M-H]-1	5409888.238	fatty acids
1,2,3,4-Tetramethyl-1,3-cyclopentadiene	C9 H14	-7.67	122.10861	123.11589	33.599	[M+H]+1	5057397.159	others
Demethylwedelolactone	C15 H8 O7	-6.14	300.02516	299.01788	29.949	[M-H]-1	4370016.81	others
Isorhamnetin	C16 H12 O7	-6.1	316.05638	315.04922	31.718	[M-H]-1	3652401.89	flavonoids
Ergosterol peroxide	C28 H44 O3	-2.85	428.32782	451.3179	49.292	[M+Na]+1	3104535.842	others
Hexadecanedioic acid	C16 H30 O4	-6.43	286.21257	285.20529	40.889	[M-H]-1	2594306.716	fatty acids
Glycyrrhizic acid	C42 H62 O16	-5.6	822.39918	821.39191	33.26	[M-H]-1	1788903.786	others
Eriodictyol	C15 H12 O6	-5.47	288.06181	287.05453	28.788	[M-H]-1	1725798.315	flavonoids
Penicillic acid	C8 H10 O4	7.56	192.04088	193.04816	28.11	[M+H]+1	920799.4102	others
